# Thermally Driven Wrinkle Realignment for Morphology-Controlled Enhancement of Dry Adhesion Performance

**DOI:** 10.1021/acsami.5c15362

**Published:** 2025-09-11

**Authors:** Yu-Fang Lai, Jui-Yuan Ho, Jun-Rong Chen, Yu-Fang Tsai, Han-Yu Hsueh

**Affiliations:** † Department of Material Science and Engineering, 34916 National Chung Hsing University, Taichung 40227, Taiwan Republic of China; ‡ Innovation and Development Center of Sustainable Agriculture (IDCSA), National Chung Hsing University, Taichung 40227, Taiwan Republic of China; § Graduate Program in Semiconductor and Green Technology, Academy of Circular Economy, National Chung Hsing University, Nantou City, Nantou 540216, Taiwan Republic of China

**Keywords:** wrinkle, interfacial release, thermally driven, dry adhesion, orientation

## Abstract

In this study, we developed a novel and scalable strategy for fabricating aligned wrinkle patterns through thermal expansion and contraction. Carbon nanotubes were incorporated into polydimethylsiloxane to enhance its (i.e., PDMS) heat transfer within the bilayer structure. This thermal activation enabled reversible wrinkle arrangement, allowing for the formation of highly ordered wrinkle morphologies through a purely physical process, without the need for vacuum conditions or complex precursor synthesis. The influences of geometric parameters (the sample aspect ratio and size) on wrinkle alignment were systematically investigated. We identified that the synergistic coexistence of flat regions and aligned wrinkle patterns enhances dry adhesion. The flat region increases the real contact area, whereas the wrinkled region provides mechanical interlocking and suppresses crack propagation. This study found that surface roughness, wrinkle morphology, and spatial wrinkle arrangement play critical roles in determining dry adhesion performance. In summary, this paper presents a facile, reversible, and industrially viable approach for generating functional surface patterns that can be employed in a wide range of emerging technologies.

## Graphical abstract

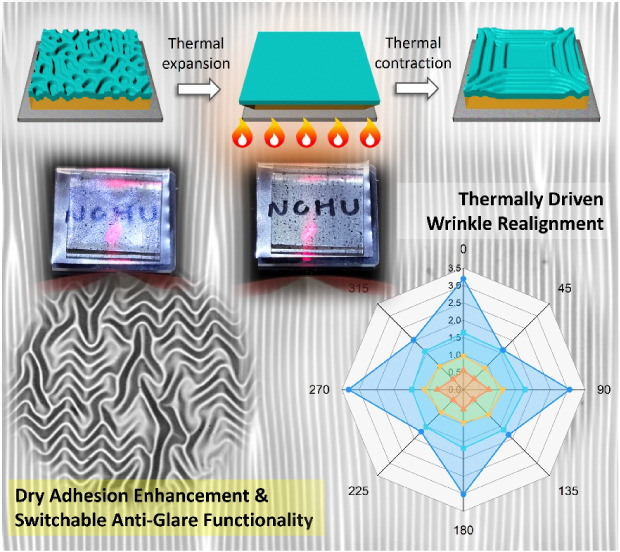

## Introduction

Surface patterning at microscale and nanoscale levels has garnered substantial scientific and technological interest because of its wide range of functional applications. Moreover, considerable research conducted in various disciplines has explored mechanical instabilities in soft materials, particularly buckling phenomena, because of their simplicity, low fabrication cost, and versatility in generating diverse surface morphologies.^1–3^ In a typical bilayer system, in which a stiff thin film is supported by a compliant substrate, buckling occurs to minimize the total potential energy when residual stresses exceed a critical threshold. These stresses can be induced by various stimuli, including thermal contraction,^4–6^ solvent swelling,^7–9^ mechanical deformation,^10–13^ capillary forces,^14,15^ and volume changes in cross-linked gels.^16,17^ Buckling patterns are common in everyday life on various surfaces, such as pruned skin (after extended soaking), aged or wrinkled skin, partially cured coatings, and deteriorated wallpaper. As a self-organizing mechanism, surface instability can enhance interfacial properties such as hydrophobicity, friction, adhesion, and effective contact area. Leveraging these attributes, buckled patterns have been applied in a broad range of fields, including tunable optical devices,^18,19^ responsive microfluidic channels,^20^ microlens arrays,^20,21^ thin-film metrology,^22–24^ dry adhesion,^25,26^ particle sorting,^27,28^ marine antifouling,^29–31^ cell alignment,^32^ flexible electronics,^33,34^ reversible devices,^35–37^ and sensors.^38,39^

Wrinkled surface morphologies can be broadly classified into three primary categories on the basis of their geometric alignment and spatial regularity: aligned wrinkles, zigzag (or herringbone) wrinkles, and random wrinkles. Aligned wrinkles exhibit periodic undulations with a well-defined orientation, and they are typically generated under uniaxial compressive stress. Their formation is governed by the interplay between the bending stiffness of the rigid capping layer and the compliance of the underlying substrate, resulting in sinusoidal patterns with predictable wavelengths and amplitudes. By contrast, zigzag wrinkles display periodic directional shifts arising from biaxial or anisotropic stress fields.^40–42^ These patterns serve as a source of secondary instability for releasing additional strain energy to that released through the primary buckling mode. Random wrinkles lack long-range order and have irregular orientations and spacings.^4,43,44^ They are generally caused by spatially heterogeneous stress distributions, substrate imperfections, or isotropic contraction and are characterized by the absence of translational and rotational symmetry. Of the aforementioned morphologies, aligned wrinkles are particularly attractive because of their anisotropic properties and highly tunable nature. Their structural regularity and directional control can play crucial roles in diverse applications, including dry adhesives,^45^ microfluidic channels,^46^ cell alignment platforms,^47,48^ liquid crystal orientation layers,^49,50^ and phase gratings.^51^ The ability to engineer wrinkle dimensions and orientations with high precision would mean that aligned wrinkle systems have high promise for next-generation functional surfaces.

Various techniques have been developed for fabricating aligned wrinkle patterns, with the most commonly adopted approaches falling into two categories: mechanical-strain-induced wrinkling^45,52^ and template-guided wrinkling.^53–55^ With regard to mechanical-strain-induced wrinkling, Lin and Yang^52^ subjected a polydimethylsiloxane (PDMS) substrate to uniaxial stretching and then treated it with oxygen plasma to generate a thin oxidized siliceous surface layer. To enhance surface roughness and modulus contrast, they deposited silica nanoparticles on the aforementioned surface layer through dip coating. Upon releasing the prestrained PDMS, microscale aligned wrinkles were produced because of the mechanical mismatch between the stiff oxidized layer and the underlying elastomer. In contrast to mechanical-strain-based methods, template-guided methods involve directing the formation of wrinkles through spatial confinement. Whitesides et al.^55^ reported a strategy in which PDMS was soaked in a benzophenone solution and selectively exposed to ultraviolet (UV) light through an amplitude photomask, which induced localized stiffening of the polymer surface. Subsequent heating caused regions with different stiffness to expand at different rates because of differences in thermal expansion coefficients. A thin gold film was then deposited on the PDMS surface through electron beam evaporation. As the PDMS substrate subsequently cooled, the residual compressive stress in the metal layer was released, resulting in the spontaneous formation of a buckling pattern that reflected the photomask’s geometry. Critically, the spatial confinement imposed by the template effectively guided the wrinkles’ orientation and enabled the formation of highly ordered structures. Despite their effectiveness, methods for fabricating aligned wrinkle patterns have several limitations. First, large-scale fabrication through mechanical stretching is inefficient because of challenges in applying uniform stress. Second, template-based strategies often involve time-consuming prepatterning and are associated with low material compatibility. More importantly, both approaches typically require an ultrahigh-vacuum processing approach, such as physical or chemical vapor deposition, which is costly and incompatible with many soft or thermally sensitive materials; thus, the approaches have limited practical scalability.

To address the aforementioned challenges, this study developed a novel and scalable strategy for fabricating aligned wrinkle patterns through thermal expansion and contraction. Carbon nanotubes (CNTs), which have excellent thermal conductivity,^56^ were incorporated into PDMS to enhance the transfer of heat within the bilayer structure (PS/CNTs-PDMS). This thermal actuation triggered reversible wrinkle arrangement, enabling the formation of high-quality aligned wrinkle morphologies through a purely physical process, without the need for vacuum conditions or complex precursor synthesis. In addition, the influences of geometric factors, such as sample aspect ratio and sample size, on wrinkle orientation were systematically investigated. Finally, the dry adhesion performance of the realigned wrinkled structure was evaluated to demonstrate its practical utility. This study is the first to identify that the synergistic coexistence of flat regions and aligned wrinkle patterns enhances dry adhesion. The flat region effectively increases the real contact area, whereas the wrinkled region provides mechanical interlocking and suppresses crack propagation. The study determined that surface roughness, wrinkle morphology, and spatial wrinkle arrangement play critical roles in determining dry adhesion performance. The developed approach is a facile, reversible, and industrially viable route for generating functional surface patterns that can be adopted in a broad range of emerging applications.

## Results and Discussion

### Mechanism of Thermally Driven Wrinkle Reorganization under Substrate Constraints

Surfaces with randomly distributed wrinkled patterns were prepared using an approach in which dynamic-interfacial-release-induced buckling. This method relies on the kinetic release of local strain, which is facilitated by dissolution of a sacrificial layer along the edges of a multilayer composite. Thermally induced cross-linking causes the volume of the composite to decrease, driving the development and stabilization of surface buckling morphology, as thoroughly described in our previous report.^57^ The process employed in the present study is summarized as follows. A water-soluble polymer, namely poly­(vinyl alcohol) (PVA), was first spin-coated onto clean glass substrates to serve as a sacrificial layer. Polystyrene (PS) was then spin-coated onto the PVA layer, and liquid CNTs-PDMS precursors were cast on the PS film, with the PS film and CNTs-PDMS precursors serving as the stiff film and soft layer for the bilayer wrinkle system, respectively. After thermal curing was performed, the CNTs-PDMS elastomer layer was fully cross-linked, and internal contraction stress was generated during the cross-linking process and stored within the multilayer composite. The interface of the bilayer system became unstable when the internal stress of the sample was released by dissolving the sacrificial layer. Therefore, in the system, disordered wrinkles spontaneously generated to decrease the interfacial energy, thereby stabilizing the system (Scheme 1a). Subsequently, sample heating resulted in flattening of the formed wrinkled patterns because of the thermal expansion of the CNTs-PDMS elastomer within a few minutes (Scheme 1b). Because of the strong adhesion of the CNTs-PDMS elastomer to the glass substrate, the thermal expansion of the CNTs-PDMS layer was confined, resulting in a negligible volume change at the bottom of the sample, which stored the compressive stress generated during the heating process. As the sample slowly cooled to room temperature, the stored stress was released from the boundaries toward the center because of thermal contraction, resulting in the generation of highly oriented and symmetrical wrinkled patterns. The symmetrical wrinkles formed parallel to and near the sample boundaries. These oriented wrinkles were reversible and had a symmetric pattern, thus showing potential for application as a dry adhesive (Scheme 1c).

**Scheme 1:**
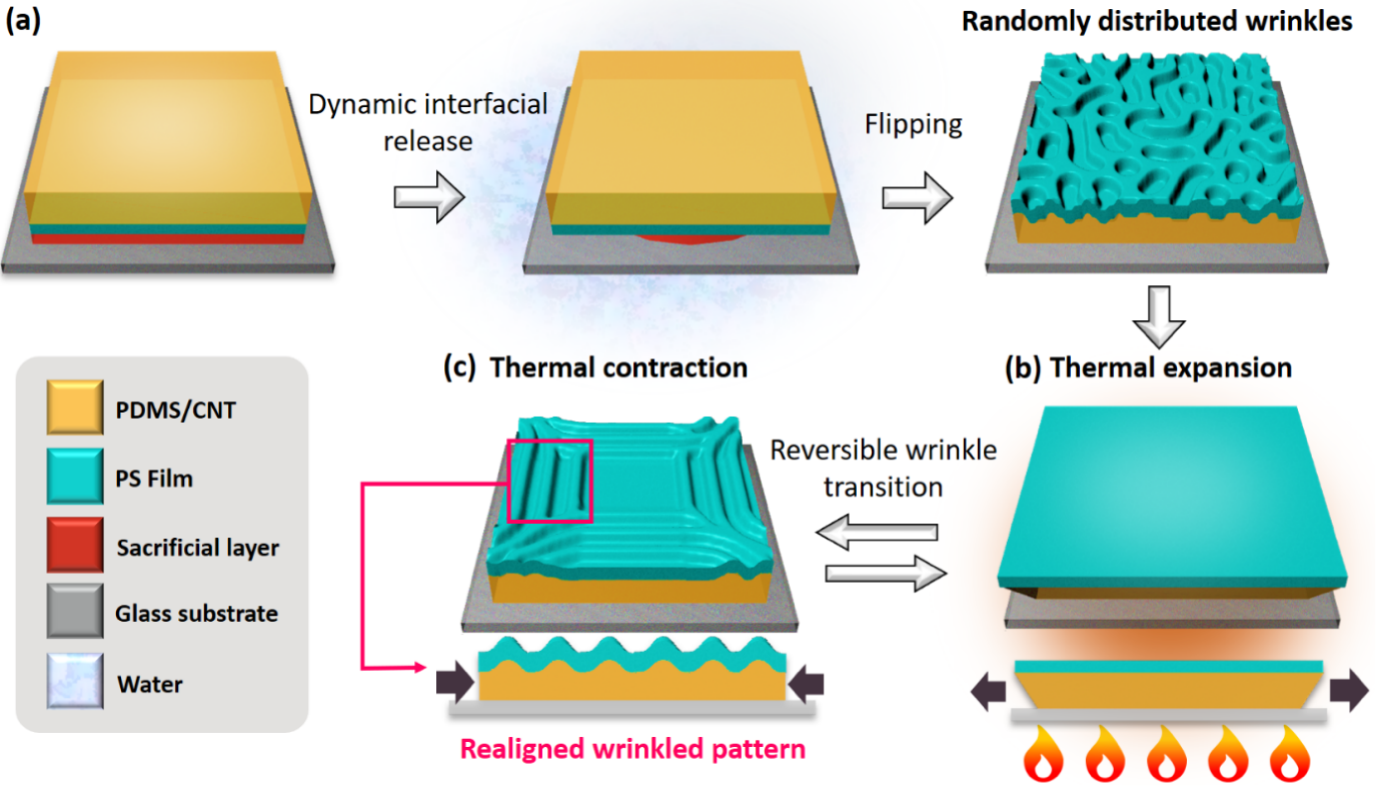
Schematic of Thermally Induced Realignment of Symmetrically Oriented Wrinkled Patterns for Dry Adhesion Surfaces[Fn sch1-fn1]

i (a) Formation of randomly distributed wrinkles through buckling induced by dynamic-interfacial-release. (b) Flattening of the formed wrinkles through thermal treatment. (c) Regeneration and realignment of highly oriented and symmetrical wrinkled surfaces during thermal contraction for dry adhesive applications. Orange, cyan, brown, and gray represent the CNTs-PDMS precursor, PS, sacrificial polymer layer, and glass substrate, respectively.

Before thermally driven wrinkle realignment, the wrinkled surface fabricated through the dynamic-interfacial-release process could be divided into three zones, as reported in our previous study.^57^ From the edge to the center of the sample, these zones comprised wrinkles perpendicular to the sample edges (edge zone); shape-induced, long-range ordered wrinkles (shape-oriented zone); and a disordered pattern of multiple concentric circles (random zone). The edge zone, shape-oriented zone, and random zone (denoted by orange, pink, and blue, respectively, in ) accounted for approximately 23.5%, 72.4%, and 4.1% of the area of each sample, respectively. After a complete heating cycle (i.e., heating followed by cooling), wrinkles were regenerated and rearranged on the sample surfaces, resulting in a different wrinkle distribution compared with that in the original state. This regeneration and rearrangement were attributable to the thermal expansion and contraction. During heating, thermal expansion caused an increase in the elastomer substrate’s volume, flattening the surface; subsequent cooling contraction then resulted in compressive stress, leading to regeneration of unstable surface morphologies. clearly shows the evolution from randomly distributed wrinkles to aligned wrinkles while the sample was confined by the underlying glass substrate. To enhance the thermal expansion efficiency of the elastomer substrate, CNTs were added to the PDMS precursor, forming a CNTs-PDMS elastomer composite. As displayed in Figure 1a, the initially apparent wrinkled patterns gradually flattened during the heating process (from 30 to 90 °C) because of the intrinsic thermal expansion property of PDMS and the enhanced thermal conductivity provided by the added CNTs. The wrinkles fully disappeared at 90 °C, with the disappearance requiring several minutes.

**Figure 1:**
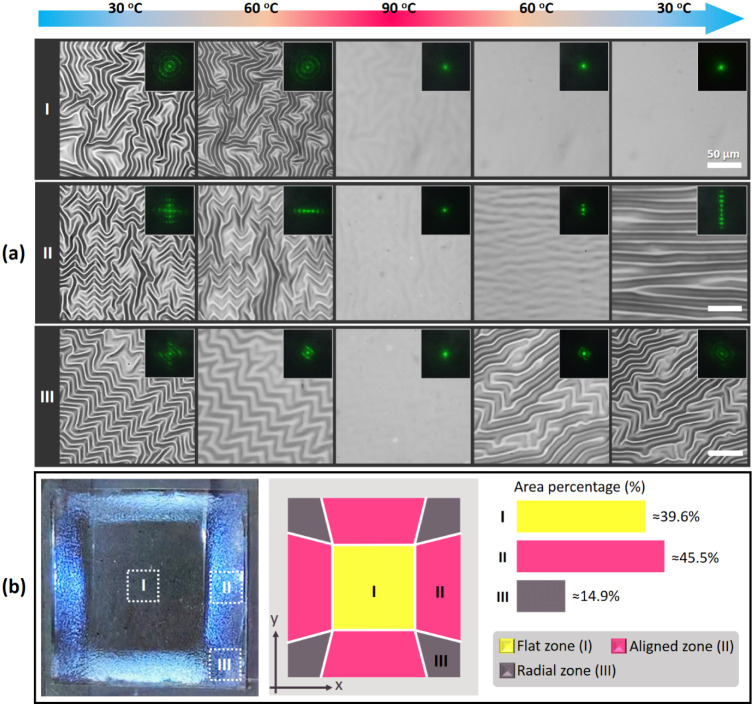
(a) Optical images of three sample zones—a flat zone (Zone I), an aligned zone (Zone II), and a radial zone (Zone III)—before and after a heating–cooling cycle. The inset images show the laser scattering patterns, with the scale bar representing 50 μm. (b) Photograph of a wrinkled sample after a heating–cooling cycle as well as an illustration showing the distribution ratios of the three zones with diverse wrinkle orientations on the surface. The yellow, pink, and gray regions represent the flat zone, aligned zone, and radial zone, respectively.

During the cooling process (from 90 to 30 °C), shrinkage of the composite elastomer generated compressive stress, which was constrained by the glass substrate, limiting the bottom of the elastomer. Thus, the composite elastomer’s overall volume did not decrease, and the compressive stress transferred to the sample surface, further promoting and stabilizing the regeneration and rearrangement of surface wrinkles. Because of the varying compressive stress across the sample surface, the regenerated wrinkled patterns could be divided into three zones: a flat zone (Zone I), an aligned zone (Zone II), and a radial zone (Zone III), which are represented by yellow, pink, and gray in Figure 1b, respectively. The varying degrees of wrinkle alignment in these regions resulted in them having distinct light reflection properties, with the resultant reflection pattern being similar to those exhibited by photonic crystals. Laser scattering was used to confirm the overall distribution of the wrinkled patterns generated through this rearrangement process (insets in Figure 1a). Zone II initially displayed wrinkles that were perpendicular to each other before heating (30 °C), producing a cross-shaped laser pattern (Figure 1a). However, after the sample had been heated to 90 °C, the laser scattering pattern appeared as a single dot, indicating a flat surface without patterns. Upon cooling the sample back to 30 °C, the laser scattering pattern transformed into a perpendicular dotted line, which represented the formation of highly oriented wrinkles in a single direction parallel to the sample edges, resulting in low reflection. Zone II covered approximately 45.5% of the total sample area. Zone III appeared at the four corners of the tested samples, where long-range radial wrinkles were formed. This zone occupied approximately 14.9% of the sample area. Because the compressive stress was influenced by adjacent edges, the stress in Zone III was complex, resulting in disordered wrinkle patterns; no notable alignment was observed before or after heating. The laser scattering pattern comprised concentric circles, indicating the existence of randomly distributed wrinkles with a defined wavelength. Zone I was located in the center of the sample, away from the edges. In this area, the compressive stress was minimal or canceled out by stresses from various directions after cooling, resulting in a flat surface morphology that exhibited clear specular reflection. Consequently, after the sample was cooled from 90 to 30 °C, the laser scattering pattern remained a single dot, indicating the absence of wrinkle formation. Zone I occupied approximately 39.6% of the total surface area. In contrast to the scenario before thermal recovery, the central region (Zone I) was a flat surface, replacing the previously random wrinkled patterns.

The volume of the elastomer substrate changed during a heating–cooling cycle; however, the shrinkage of the bottom part of the elastomer was constrained by the glass substrate. Consequently, the compressive stress generated during cooling was transferred to the sample surface, leading to rearrangement of surface wrinkles. To verify this hypothesis, CNTs-PDMS elastomer composites without a glass substrate (i.e., free-standing) were subjected to a heating–cooling cycle (Figure 2a). During the heating process (from 30 to 90 °C), the morphological transition was similar to that of the samples tested on a glass substrate. Specifically, as the temperature increased, the wrinkles gradually disappeared, resulting in a flat surface without any residual wrinkled patterns. However, after cooling (from 90 to 30 °C), the original wrinkled patterns were regenerated—no reorganization occurred. In the absence of a glass substrate, the CNTs-PDMS elastomer composites exhibited isotropic thermal expansion and contraction, which caused the wrinkles to emerge in their original distribution, similar to a memory effect. Figure 2b shows the wavelengths and amplitudes of the wrinkles generated on the test samples during the heating–cooling cycle. The amplitude decreased as the temperature was increased and then increased as the temperature was decreased; however, the wavelength was the same regardless of the temperature. Thus, although the wavelengths and amplitudes of the wrinkles were recoverable before and after heating, the constraint provided by the glass substrate was critical to the regeneration, reorganization, and realignment of the wrinkled surface.

**Figure 2:**
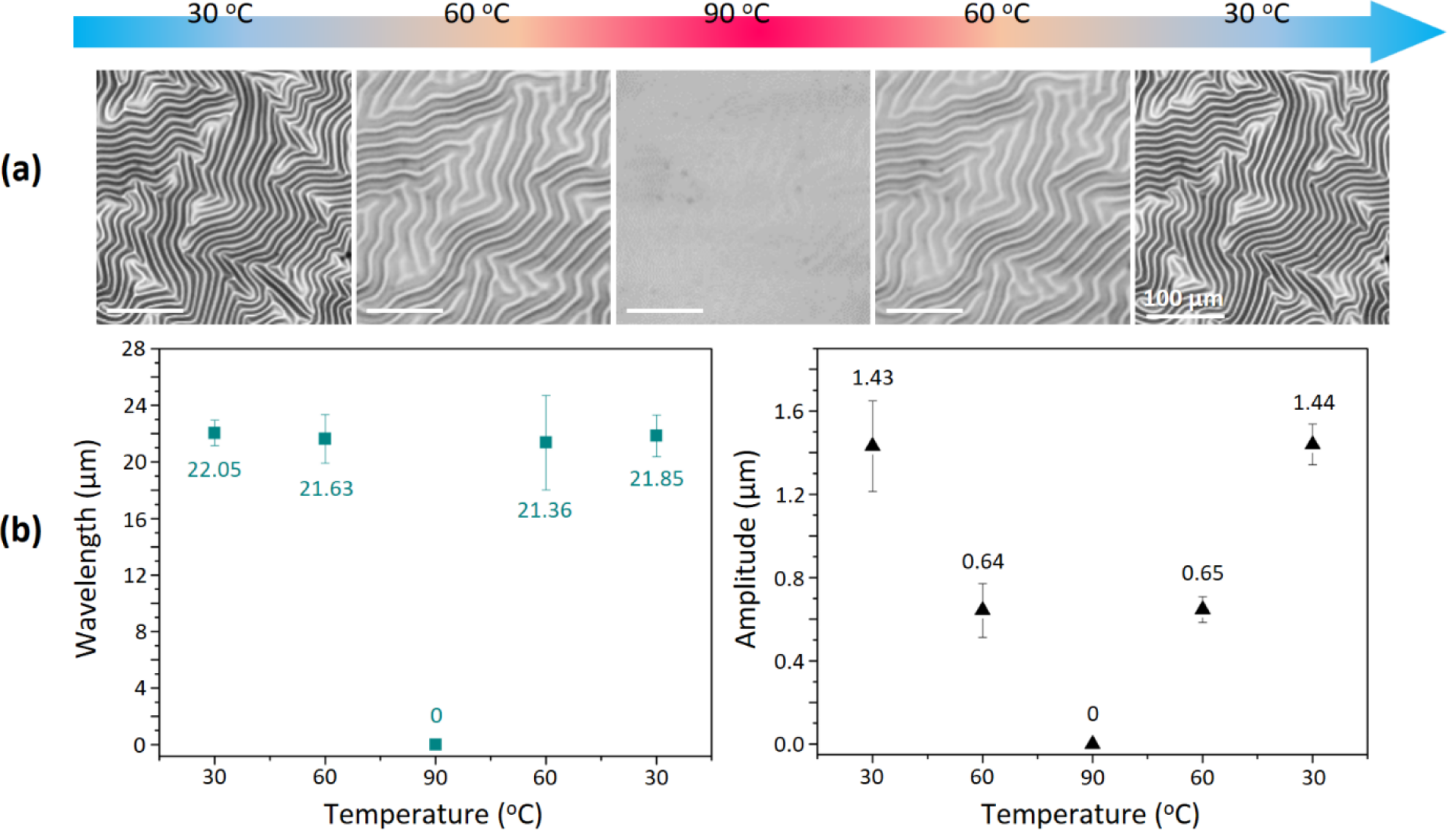
(a) Confocal images of PS/CNTs-PDMS elastomer composites without a glass substrate during a heating–cooling cycle. The scale bar represents 100 μm. (b) Corresponding wavelengths and amplitudes of the wrinkles generated during the heating–cooling cycle.

### Geometric Control of Thermally Induced Wrinkle Alignment

Because the compressive stress was attributable to the recontraction of the sample after heating, this stress might have been influenced by the sample size. Specifically, larger samples can result in greater stress accumulation, which affects the wrinkle distribution. To determine the effect of sample size on the formation of aligned wrinkles, we prepared several square samples with differing dimensions for use in thermal expansion and contraction experiments. Figure 3a displays the realigned areas of the samples (i.e., Zone II, represented in pink) and the area percentage of the realigned area for square samples with different side lengths. When the side length was less than 1.5 cm, the compressive stresses generated from different edges propagated across the entire sample surface, resulting in these stresses interacting with each other. Consequently, the produced wrinkles were randomly distributed across the entire sample surface, with distinct zones not being formed after the reorganization process. Thus, no realigned patterns formed, and the Zone II area accounted for 0% of the sample. When the side length was 1.5 cm, the ratio of the realigned area (Zone II) was at its highest value of 80%, with the generation of aligned areas being effectively maximized. However, when the side length exceeded 1.5 cm, the proportion of Zone II decreased as the side length was increased, although the overall area of Zone II increased. This decrease in proportion was likely caused by the sample’s considerable size, which made it difficult for the contraction stress to propagate uniformly throughout the sample. Consequently, a larger flat region (Zone I) formed at the center of the sample. Thus, wrinkles were confined to smaller areas, leading to a decrease in the Zone II proportion. On the basis of the aforementioned results, 1.5 cm was identified as the optimal side length for maximizing wrinkle realignment in the CNTs-PDMS elastomer system (precursor-to-cross-linker ratio of 30:1). In general, coating size is a critical parameter that must be carefully controlled for the effective realignment of wrinkle patterns in material systems.

**Figure 3:**
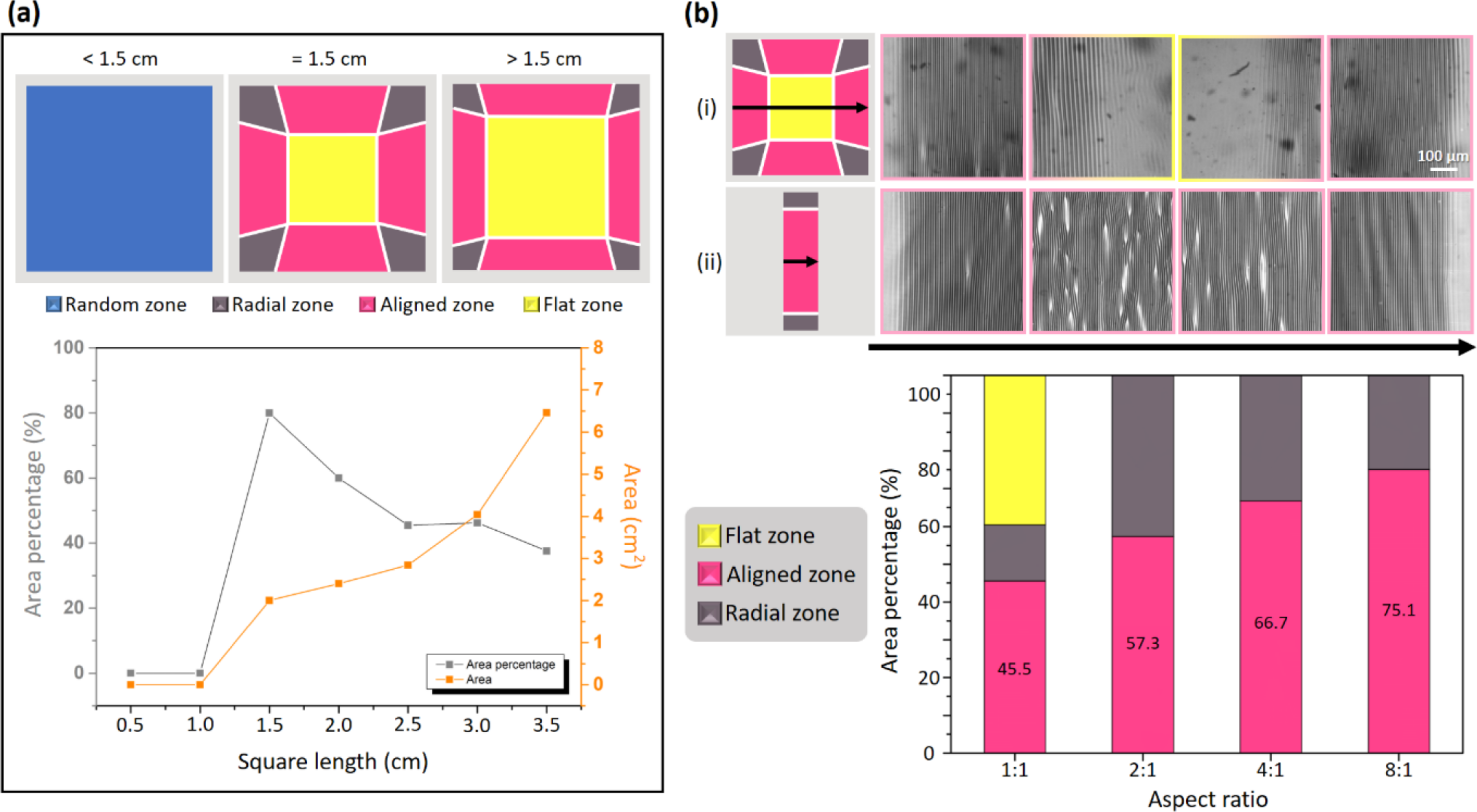
(a) Images and ratios of the aligned areas in square samples with different side lengths. The illustrations from left to right show samples with square lengths of <1.5, 1.5, and >1.5 cm. (b) Top: Confocal microscopy images showing the wrinkle distribution from the left edge to the right edge for (i) square and (ii) rectangular samples. Bottom: Area percentages of flat, aligned, and radial zones in samples with different aspect ratios.

In addition to sample size, sample aspect ratio played a crucial role in determining the distribution and extent of the aligned wrinkle zones. When the sample had a symmetric shape (a square), contraction stresses from different directions counteracted each other at the center during the heating and cooling processes, resulting in a flat central region, namely Zone I [Figure 3b­(i)]. By contrast, when the sample had an asymmetric shape (a rectangle), contraction stresses from the longer sides propagated more effectively across the sample without being fully counteracted by stresses from the shorter sides. This asymmetry suppressed the formation of Zone I and promoted large-area wrinkle alignment. As shown in Figure 3b­(ii), in rectangular samples, oriented wrinkles emerged from one edge and extended uniformly across the entire surface. Aligned wrinkle patterns that completely covered the sample were generated by simply adjusting the samples’ geometry. As illustrated in the bar chart at the bottom of Figure 3b, when the aspect ratio was increased from 1:1 to 8:1 (long side:short side), Zone I disappeared, and the proportion of oriented wrinkles gradually increased, reaching to 75.1%. When the difference in side lengths was sufficiently large, uniform wrinkle orientation was achieved across the entire sample surface. Notably, our results indicate that the effect of sample shape on the formation of aligned wrinkles is stronger than that of sample size, with the effect of sample size becoming negligible in samples with anisotropic geometry. These findings indicate that controlling both samples’ size and aspect ratio is an effective and straightforward strategy for optimizing wrinkle alignment and achieving the desired surface morphology. Apart from size and aspect ratio, wrinkle realignment can also occur in samples with different geometries. We examined a circular specimen before and after a heating–cooling cycle (). Similar rearrangement behavior was observed even for the circular geometry: after thermal cycling, the center of the sample became a flat region, while the edge exhibited aligned wrinkles. In contrast to square samples, no radial zones were present in the circular specimen due to the absence of corners. These results support the conclusion that wrinkle alignment during the heating–cooling cycle is primarily governed by substrate-imposed edge-constrained shrinkage. Consequently, the shape and extent of the flat central region reflect the overall geometry of the specimen.

According to the theoretical wrinkling model for bilayer systems, the thickness of the elastomer substrate does not influence the structural characteristics of generated wrinkles.^58^ This model aligns with our findings. As shown in , the wavelengths and amplitudes of the wrinkles before (solid line) and after (dashed line) thermal realignment did not differ substantially. However, sample thickness was a key factor affecting the distribution ratio of the formed oriented wrinkles. As displayed in , under the same heating duration and heated area, when the elastomer thickness was increased from 1.0 to 4.0 mm, the proportion of the aligned wrinkled zone considerably increased (i.e., the proportion of the flat zone decreased) from 8.4% to 52.3% (aligned zone, marked in pink in ). Moreover, a small random zone emerged, accounting for approximately 5.5% of the sample area. This result is attributable to thicker samples accumulating more stress during the thermal expansion–contraction process. During cooling, the contraction stresses propagating from the sample edges toward the center increased considerably. As the range of stress influence expanded, areas farther from the sample boundaries were more strongly affected by contraction stresses, leading to the formation of additional oriented wrinkles.

High stability of wrinkled patterns is critical for various applications. To confirm the stability of the realigned wrinkles, the wrinkled samples were stored for a long-term period under ambient conditions (Figure 4a). Although the realigned wrinkles exhibited slightly smaller wavelength and amplitude after thermal treatment, these parameters were stable after 30 days of sample storage. This result indicated that the thermally realigned wrinkles did not dissipate over time, thus confirming their stability. Furthermore, the thermally induced realigned wrinkled patterns exhibited repeatability and memory effects. The test samples were subjected to multiple heating–cooling cycles (Figure 4b). The samples initially exhibited a disordered wrinkled surface and then showed thermally induced wrinkle realignment. After the first heating step, the surface wrinkles had disappeared because of thermal expansion; subsequent cooling led to regeneration of aligned wrinkles. In subsequent heating–cooling cycles, the samples consistently exhibited stable wrinkle rearrangement under temperature changes, with only minor variations observed in the wavelength and amplitude of the wrinkles. The generated wrinkles exhibited remarkable thermal responsiveness, being repeatedly rearranged under heating–cooling cycles without exhibiting geometric degradation over time. Moreover, the realigned wrinkle patterns exhibited a memory effect; the wrinkle patterns generated in the first heating–cooling cycle were identical to those produced in the subsequent heating–cooling cycles (see the confocal microscopy images in the inset of Figure 4b). This high structural stability makes the sample material particularly promising for applications requiring reliable thermal responsiveness over long durations and multiple operational cycles.

**Figure 4:**
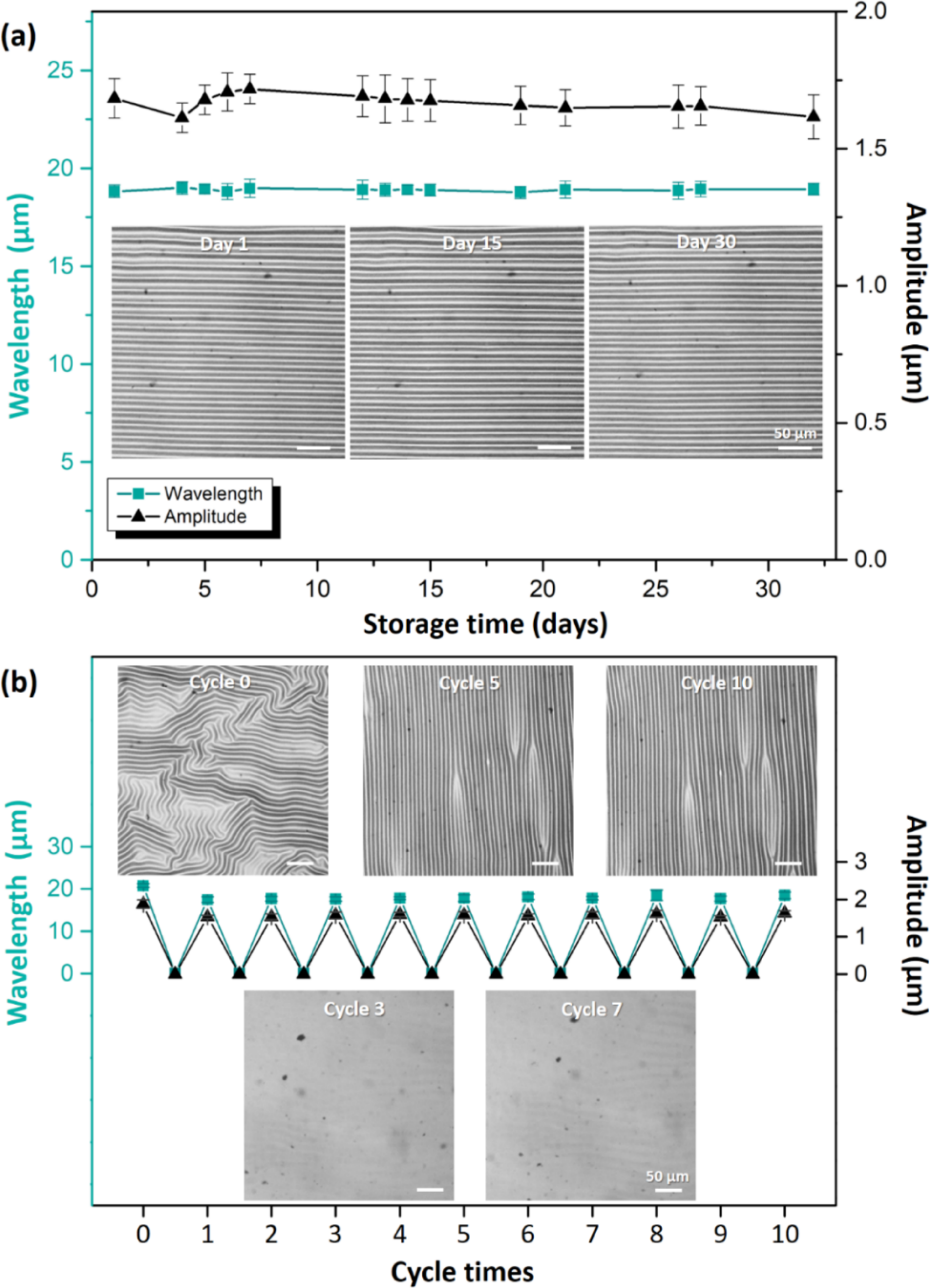
(a) Wavelengths (green line with square markers) and amplitudes (black line with triangular markers) of the realigned wrinkles versus storage time. (b) Variations in wrinkle wavelength (green line with square markers) and amplitude (black line with triangular markers) over multiple cyclic heating and cooling processes. Inset: confocal microscopy images of the surface morphologies of a sample. All results were verified through five experiments.

### Effects of Wrinkle Alignment on Adhesion Performance and Load Directionality

Aligned wrinkled structures can be employed in various applications, including the enhancement of dry adhesion.^45^ Wrinkled surfaces’ enhancement of dry adhesion has attracted considerable attention because of its potential in biomimetic adhesives and flexible devices. Wrinkling creates periodic surface topographies that introduce roughness, which fundamentally alters the interfacial contact mechanics between an elastomeric substrate, such as PDMS, and its counter surfaces. Compared with flat surfaces, surfaces with wrinkled structures offer greater effective compliance, enabling stronger conformal contact under applied load, which results in a larger real contact area for an identical nominal contact area. Furthermore, wrinkled surfaces facilitate mechanical interlocking by allowing the counter surface to physically interpenetrate or lock within the wrinkle valleys; this interlocking considerably increases the resistance of these surfaces against shear and normal forces. In addition to these mechanical effects, wrinkled surfaces can hinder the propagation of cracks initiated by stress during dry adhesion;^59^ the surface undulations act as geometric barriers that suppress peeling and delay interfacial separation. Although the ability of wrinkled structures to enhance dry adhesion has been widely studied, with the main focus on surface roughness and amplitude, the effects of overall wrinkle patterns and their spatial distribution on adhesion performance have received comparatively little attention.

To evaluate the effect of the spatial wrinkle distribution on adhesion performance, this study programmed a piezocontrolled linear actuator to bring the test sample into contact with a clean glass slide. A fixed preload was then applied to the sample by driving the actuator at a constant displacement rate, ensuring consistent contact conditions. After the target compression was reached, the actuator was reversed at a controlled speed to initiate detachment. Throughout this process, the force response was continuously recorded, capturing the transition from compression to separation. The maximum adhesion force typically occurred at the point when the sample detached from the glass surface. The normal force was determined by vertically detaching the upper glass slide from the wrinkled surface. By contrast, the shear force was measured by laterally sliding the glass slide along the sample surface. During adhesion, the apparent contact area corresponds to the wrinkle crests, and both the wrinkle wavelength and amplitude remain essentially unchanged (i.e., no collapse of the topography). These observations confirm that contact occurs on the wrinkle surfaces rather than on flattened regions (). However, direct measurement of the total contact area over a large wrinkled surface is challenging. Therefore, we employed ImageJ for image analysis to estimate the total contact area. Confocal microscopy images were first converted into grayscale images, following which a threshold was applied to generate binary images. This process enabled the identification and labeling of contact regions () and thus calculation of the contact area. Contact areas of 135.1, 160.5, 125.5, and 400.0 mm^2^ were obtained for PS/CNTs-PDMS samples with random wrinkles, realigned wrinkles, aligned wrinkles, and no wrinkles (a flat surface), respectively ().

The adhesion performance of the aforementioned four PS/CNTs-PDMS test samples was systematically evaluated through normal and shear force measurements (Figure 5a) as well as directional shear adhesion tests (Figure 5b). Of the four samples, the flat sample had the largest contact area because of its smooth and continuous surface; however, it did not result in the highest dry adhesion force. These results indicate that although wrinkled structures reduce the overall contact area because of their complex topography, they enhance the adhesion force through interlocking effects. The dry adhesion performance of the wrinkled samples correlated with their contact areas. Of the four samples, the sample with realigned wrinkles exhibited the highest normal force (9.02 N/cm^2^) and shear force (3.20 N/cm^2^), considerably outperforming the samples with random and aligned wrinkles in terms of normal and shear adhesion. This superior performance of the sample with realigned wrinkles was attributable to its unique geometric configuration, which combined roughness with concentrated edge effects. The central flat region of the sample resulted in it having a larger overall contact area compared with the other wrinkled samples. Moreover, the wrinkle pattern of the sample, which was parallel to its edges, improved its mechanical stability and suppressed the propagation of cracks in the sample under loading. Consequently, the sample with realigned wrinkles led to a higher adhesion force than did the flat sample. The coexistence of flat and wrinkled regions in the sample with realigned wrinkles promoted localized energy dissipation and hindered crack propagation, reducing the likelihood of early detachment.

**Figure 5:**
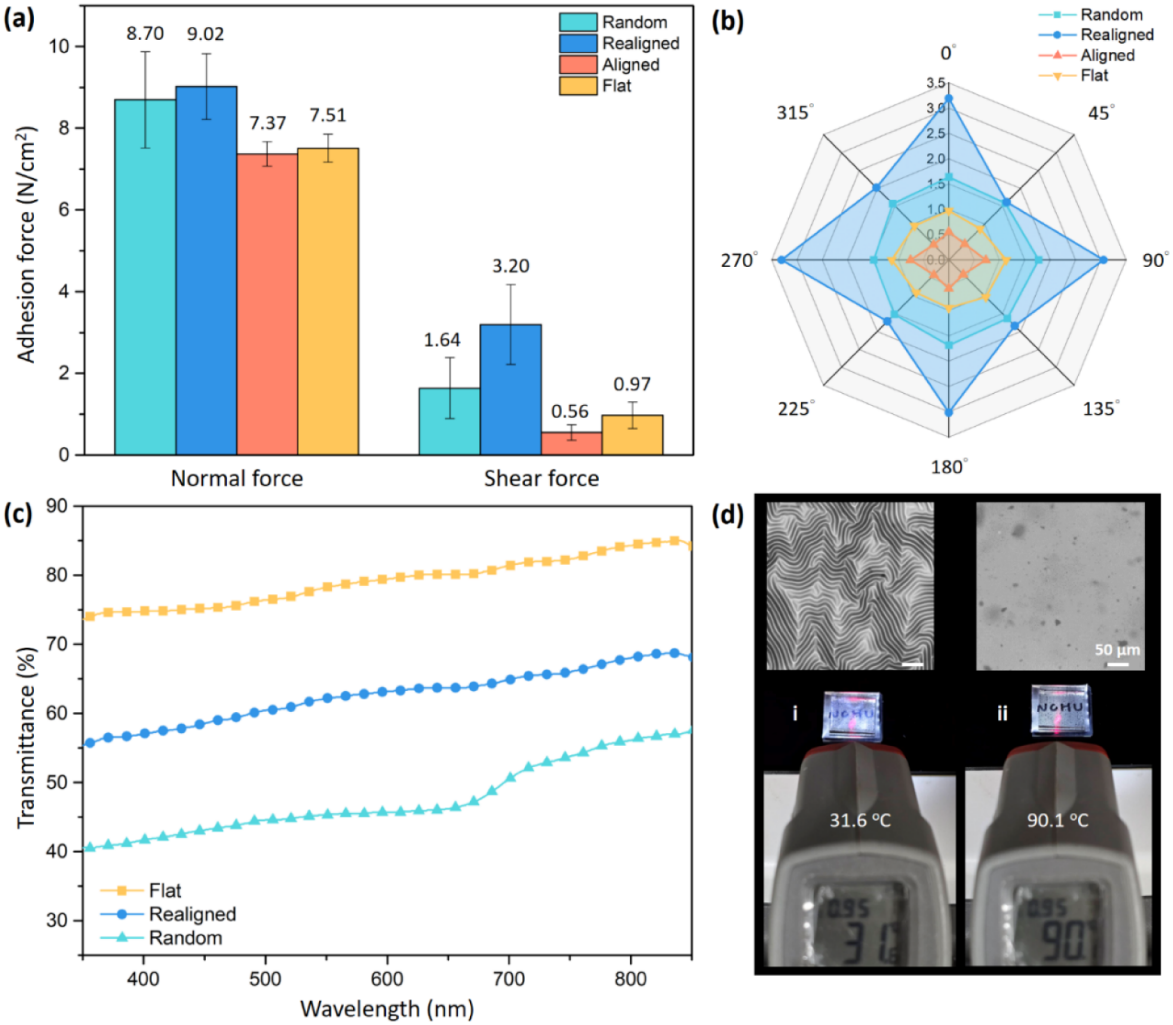
(a) Normal and shear adhesion forces of PS/CNTs-PDMS samples with various surface morphologies, namely randomly arranged wrinkles, realigned wrinkles, aligned wrinkles, and a flat structure (no wrinkles). (b) Shear adhesion performance of the samples in different directions. (c) Transmittance spectra of various surface morphologies (i.e., flat surface, realigned wrinkles, and random wrinkles) fabricated from PS/CNTs-PDMS. (d) Photographs and confocal microscopy images showing the morphology of the sample (i) at room temperature and (ii) under heating condition.

Although the sample with random wrinkles exhibited slightly lower overall dry adhesion performance than did that with realigned wrinkles, this performance was still respectable, with the corresponding normal and shear forces being 8.70 and 1.64 N/cm^2^, respectively. The adhesion performance of the sample with random wrinkles was attributable to its irregular contact architecture, which facilitated extensive mechanical interlocking but did not offer the organized directional support provided by realigned wrinkles. The randomly distributed peaks and valleys in this sample created a diverse set of contact points, allowing the sample surface to resist shear forces from multiple directions. However, the absence of long, continuous wrinkles resulted in this sample having lower overall shear resistance than that of the sample with realigned patterns. Of the wrinkled samples, that with aligned wrinkles (aligned in a single direction) had the weakest normal (7.37 N/cm^2^) and shear (0.56 N/cm^2^) adhesion. This poor performance was attributable to the highly regular, linear alignment of the wrinkles, which hindered effective interlocking under shear loading and reduced the ability of the sample surface to distribute compressive stresses. Consequently, the aforementioned sample could not compensate for the advantage resulting from the flat sample’s larger contact area, leading to it having overall lower dry adhesion performance than the flat sample did. Alignment of wrinkles in a single direction primarily supports unidirectional load transfer, making it less effective for applications requiring multidirectional stability.

Directional shear tests (Figure 5b) further highlighted the distinct anisotropic behaviors of the wrinkled samples. The samples with realigned and random wrinkles exhibited high multidirectional shear resistance because these wrinkles enabled the transfer of shear load in multiple directions. Notably, the directional shear behavior of the sample with realigned wrinkles was influenced by its four-sided geometry (square shape), resulting in peak shear forces occurring approximately every 90°, corresponding to the alignment of its sides. If this sample was redesigned to have circular geometry, a concentric wrinkle pattern would form, potentially enabling strong omnidirectional adhesive performance. The sample with aligned wrinkles exhibited considerable anisotropy, with pronounced peaks at angles corresponding to the alignment directions of the wrinkles (e.g., 90° and 270°). This highly directional behavior limits the effectiveness of aligned wrinkles in applications requiring stable adhesion across multiple directions, highlighting the trade-off between alignment precision and multidirectional stability. Overall, the results indicate that surfaces with realigned wrinkles, which have a balanced combination of strong normal and shear adhesion, are versatile candidates for multifunctional adhesive applications, particularly those in which multidirectional stability and robust load distribution are critical. Because of the presence of a PS capping layer, the PS/CNTs-PDMS wrinkled surfaces did not exhibit stronger dry adhesion than that exhibited by certain previously reported systems, such as those with polythiophene nanotubule^60^ or CNTs.^61^ However, the primary focus of this study was to investigate the effects of wrinkle morphology and distribution on dry adhesion performance. For applications requiring high dry adhesion strength, realigned wrinkle patterns can be combined with more suitable materials, such as epoxy, to achieve better adhesion performance.

In addition to the adhesive performance, the optical transmittance of the samples was also evaluated to explore how wrinkle morphology affects light transmittance through the surface. As shown in Figure 5c, within the visible wavelength range, the flat sample exhibited the highest transmittance, whereas the sample with randomly arranged wrinkles showed the lowest. This indicates that the arrangement of surface wrinkles can significantly influence the optical transparency of the samples. This effect is also visually evident under different thermal conditions (Figure 5d). At room temperature, the sample with randomly distributed wrinkles presents a matte appearance, making it difficult to clearly see the text under the sample. However, upon heating to approximately 90 °C, the wrinkles were erased due to thermal expansion, resulting in a dramatic increase in transparency and allowing the background pattern to become clearly visible. The results demonstrate that the presence and configuration of surface wrinkles can effectively reduce glare by scattering incident light, which may be useful for developing antiglare coatings with temperature-responsive properties.

## Conclusions

In conclusion, this study developed a novel strategy for fabricating aligned wrinkle patterns through thermal rearrangement. Upon heating and subsequent cooling, the generated wrinkles spontaneously reorganize, resulting in the formation of a unique configuration characterized by a flat central region surrounded by highly ordered wrinkle patterns oriented parallel to the sample edges. This morphology was hypothesized to be attributable to the confinement effect imposed by the underlying glass substrate during the cooling phase, which directs the stress distribution and wrinkle orientation. To validate this hypothesis, control experiments were performed in the absence of a rigid substrate. The experimental results confirmed that without confinement, the wrinkles retained a disordered, randomly distributed pattern even after a complete heating–cooling cycle. Moreover, we systematically investigated the influences of geometric parameters on wrinkle formation, finding that a sample’s size and aspect ratio substantially affect the extent of wrinkle rearrangement. Specifically, samples that are too small tend to exhibit uniform propagation of shrinkage stress, which prevents the formation of a flat central zone. Conversely, samples that are excessively large restrict the propagation of shrinkage stress, which reduces the area of alignment. Through optimization, we identified the combination of sample size, sample aspect ratio, and sample thickness that maximized the size of the region with aligned wrinkles. In addition, the wrinkled PS/CNTs-PDMS samples were found to exhibit remarkable stability and reusability after multiple heating–cooling cycles. In particular, the thermally driven wrinkle realignment approach can be applied to other material systems. As long as the bottom soft layer possesses a sufficiently high thermal expansion coefficient and its lateral contraction is constrained by the supporting substrate, aligned wrinkles can be produced. For instance, the stiff PS layer was replaced with poly­(methyl methacrylate) (PMMA) to form PMMA/PDMS bilayer wrinkles. After a heating–cooling cycle, aligned wrinkles again emerged (), similar to those observed in the PS/PDMS bilayer system. In addition, the PDMS elastomer was replaced with epoxy, and after thermal cycling, the rearranged PS/epoxy wrinkles were formed (). These experiments demonstrate that the thermally driven wrinkle realignment process is indeed generalizable and can be applied to a variety of material systems. The results of this study indicate that samples with both a flat region and a wrinkled region containing thermally realigned wrinkled structures effectively integrate the advantages of a large contact area and mechanical interlocking. This dual-feature design offers a potential route for the development of multifunctional dry adhesive surfaces with robust, multidirectional load resistance.

## Experimental Section

### Materials

Single-walled CNTs (≥95% carbon basis) were purchased from Sigma-Aldrich. Liquid PDMS mixtures were prepared using a Dow Corning Sylgard 184 elastomer kit, with the precursor-to-cross-linker ratio being 30:1. The stiff layer of the bilayer wrinkle system was made from PS with a molecular weight of 260 000 g/mol (Thermo Fisher Scientific). PS was dissolved in toluene to form 3 wt % solution for the spin-coating process. PVA with a molecular weight of 118,000–124 000 g/mol was purchased from First Chemical Works and prepared as a 3 wt % solution in deionized water.

### Preparation of CNTs-PDMS Nanocomposites

To ensure uniform dispersion of CNTs in a PDMS mixture, 48 g of PDMS precursor and 24 mg of CNTs were mixed with 60 mL of toluene and subjected to ultrasonication for 12 h. The solution was then left at room temperature for 2 h to allow aggregated CNTs to settle. Subsequently, the upper, well-dispersed portion of the solution was collected and heated at 120 °C for 2 days under magnetic stirring to completely remove residual toluene and maintain the suspension of CNTs. After toluene removal, a fluid PDMS precursor with well-dispersed CNTs was obtained (CNTs-PDMS).

### Fabrication of Wrinkled Patterns Through a Dynamic Interfacial Release Process

Glass slides (2.5 cm × 2.5 cm) were cleaned by subjecting them to ultrasonic treatment in acetone for 10 min and subsequently rinsing them with acetone, ethanol, and deionized water. The slides were then subjected to UV–ozone treatment for 10 min to render their surface slightly hydrophilic. For the sacrificial layer, PVA solution was spin-coated onto the UV–ozone-treated glass slides at 3000 rpm for 30 s. Subsequently, PS solution was spin-coated onto the PVA-coated slides at 3000 rpm for 60 s to produce the stiff layer of the bilayer wrinkle system. A total of 30 g of fluid CNTs-PDMS precursor was mixed with 1 g of curing agent, and the mixture was then cast onto PS/PVA/glass substrates in a 10 cm^2^ plastic Petri dish to serve as the soft substrate. The samples were degassed at room temperature for 1 h and then cured in an oven at 70 °C for 24 h, following which they were cooled to room temperature (∼25 °C). The composite samples were soaked in deionized water for 12 h to dissolve the PVA layer, resulting in their detachment from the glass slide. Consequently, CNTs-PDMS elastomer substrates coated with PS wrinkled patterns were obtained.^57^ In this study, the bilayer system is represented as PS/CNTs-PDMS.

### Thermally Induced Realignment Wrinkled Patterns

The wrinkled samples produced after a dynamic-interfacial-release process were flipped and placed on clean glass slides, with the wrinkled surface facing upward. The samples on the glass substrates were then placed on a heating stage. The samples were heated to 90 °C at a rate of 10 °C/min until the surface wrinkles disappeared, following which the samples were cooled to room temperature at a rate of 10 °C/min.

### Measurement of Normal and Shear Forces

Normal and shear forces were measured using a digital force gauge (HF-50, ALGOL) in combination with an automatic vertical servo stand (JSV-H1000, JISC). All samples used for the normal and shear force measurements had dimensions of 2 cm × 2 cm. Random wrinkles were generated through the dynamic-interfacial-release process; upon releasing the accumulated stress, the surface exhibited disordered wrinkle morphologies. As described in this study, aligned wrinkles can be obtained from rectangular samples. However, achieving a square specimen fully covered with aligned wrinkles via thermally induced rearrangement is challenging. To address this, we employed a mechanically induced method: first, a PS film was floated on water, followed by the gentle attachment of a uniaxially prestretched PDMS layer onto the PS film. The prestrain was then gradually released, producing a square sample fully covered with aligned wrinkles. For fabricating a flat PS/PDMS sample, we used a higher-concentration PS solution to obtain a thicker PS film, which suppressed buckling. The PS film was floated on water and laminated with an unstretched PDMS bulk, resulting in a flat surface without wrinkles. The wrinkled PS/CNTs-PDMS was stuck to a glass slide by using epoxy. Each test sample was placed on the stage of the servo stand. A preload of 3 kgf was applied to ensure complete contact between the sample and the glass slide. Subsequently, the sample was separated from the glass slide at a constant speed of 5 mm/min to measure the adhesion force. The displacement–force relationship was recorded using the JISC SOP-EG1 software program.

### Characterization

UV–ozone irradiation (UVGL-25 Compact UV Lamp) was employed to modify the surface of the glass slides and render them hydrophilic. The produced buckling morphologies were observed using a laser scanning confocal microscope (VK-X1000 series, Keyence Corporation of America) and an optical microscope (ECLIPSE ME600, Nikon). Moreover, a self-developed laser scattering system (532 nm) was employed to analyze the produced laser scattering patterns.

## Supplementary Material





## Notes

Confocal images of a wrinkled surface fabricated through a dynamic-interfacial-release process before heating (Figure S1); optical micrographs and photographs of a circular sample before and after a heating–cooling cycle (Figure S2); the wavelengths, amplitudes, and distribution ratios of wrinkles produced on CNTs-PDMS substrates with different thicknesses (Figure S3); optical micrographs of the interfacial contact between PS/PDMS wrinkled surfaces and a flat glass slide, showing before adhesion and under adhesion (Figure S4); and binary images obtained through thresholding and the contact areas of surfaces with random wrinkles, realigned wrinkles, aligned wrinkles, and no wrinkles (i.e., a flat surface; Figure S5); confocal images of PMMA/PDMS and PS/epoxy wrinkled surface after a heating cycle (Figure S6)­(PDF)
In situ video recording of wrinkled surface during a heating–cooling cycle (Movie S1)­(MP4)
