# Supplementary material for: Thermally Driven Wrinkle Realignment for Morphology-Controlled Enhancement of Dry Adhesion Performance
Source: ACS Appl Mater Interfaces. 2025 Sep 11;17(38):54314–24. doi: 10.1021/acsami.5c15362 (PMC12464901; doi:10.1021/acsami.5c15362)
Supplement: Supplementary file 1 [file am5c15362_si_001.pdf]

Supporting Information

Thermally Driven Wrinkle Realignment for Morphology-Controlled Enhancement of Dry Adhesion Performance

*Yu-Fang Lai, Jui-Yuan Ho, Jun-Rong Chen, Yu-Fang Tsai, and Han-Yu Hsueh**

[*] Prof. Dr. H.-Y. Hsueh
Department of Material Science and Engineering, National Chung Hsing University, Taichung 40227, Taiwan, Republic of China.
Innovation and Development Center of Sustainable Agriculture (IDCSA), National Chung Hsing University, Taichung 40227, Taiwan, Republic of China.
Graduate Program in Semiconductor and Green Technology, Academy of Circular Economy, National Chung Hsing University, Nantou City, Nantou County 540216, Taiwan, Republic of China.
E-mail: hyhsueh@nchu.edu.tw

Ms. Y.-F. Lai, Ms. J.-Y. Ho, Mr. J.-R. Chen, Ms. Y.-F. Tsai
Department of Material Science and Engineering, National Chung Hsing University, Taichung 40227, Taiwan, Republic of China.

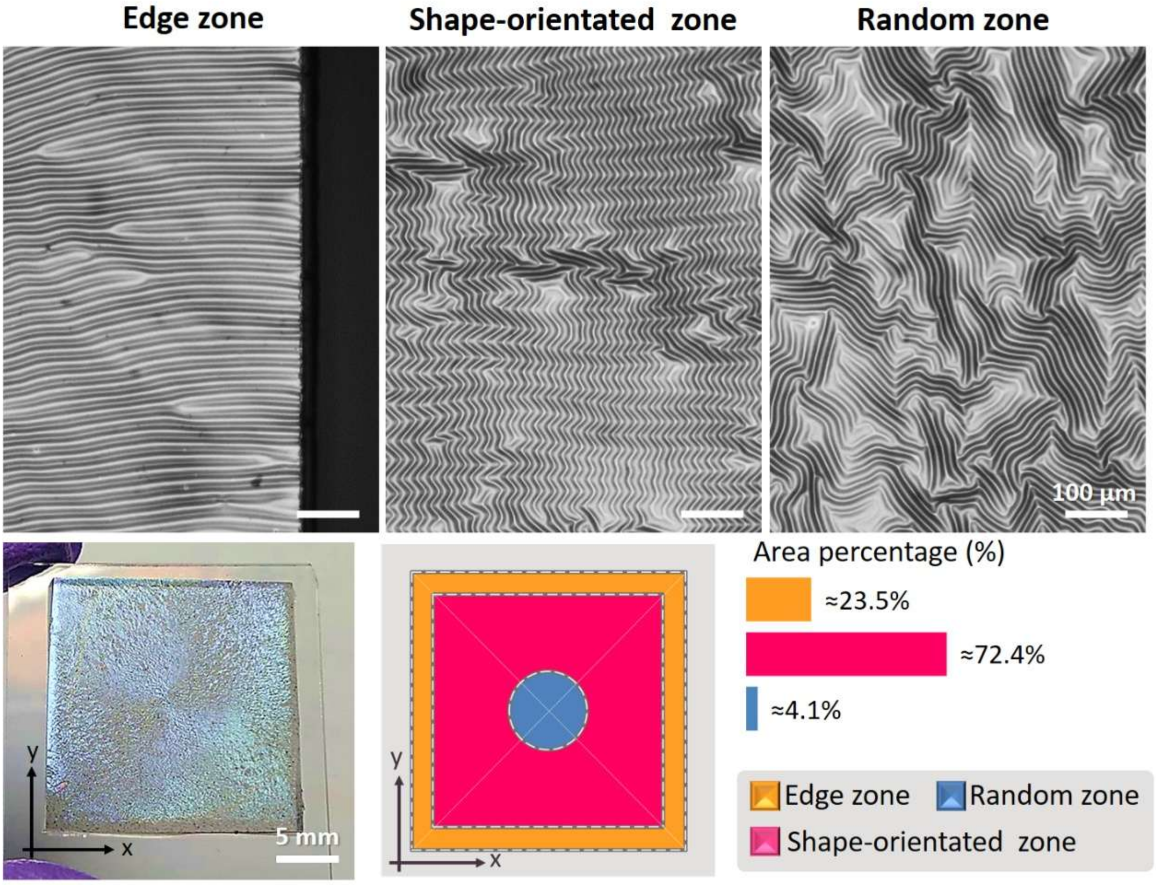

Figure S1. Confocal images of a wrinkled surface fabricated through a dynamic-interfacial-release process before heating. Top: from left to right are edge zone, shape-oriented zone, and random zone, respectively. Bottom: photograph of the wrinkled surface and the distribution ratios of the three zones with diverse wrinkle orientations across the surface, the orange, pink, and blue regions correspond to the edge zone, shape-oriented zone, and random zone, respectively.

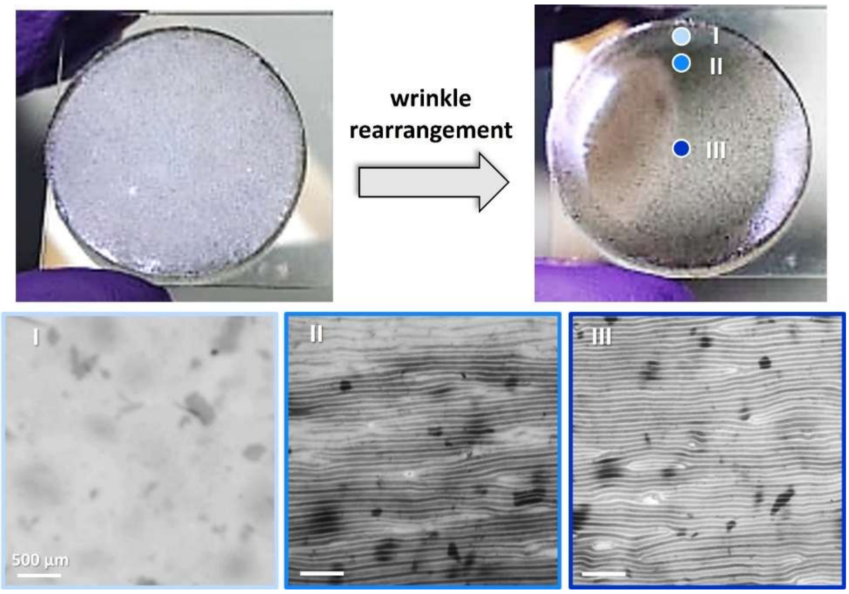

Figure S2. Optical micrographs (top) and photographs (bottom) of a circular sample before and after a heating–cooling cycle. Regions I, II, and III denote the corresponding positions from the outer edge toward the center. Scale bars represent 500 µm.

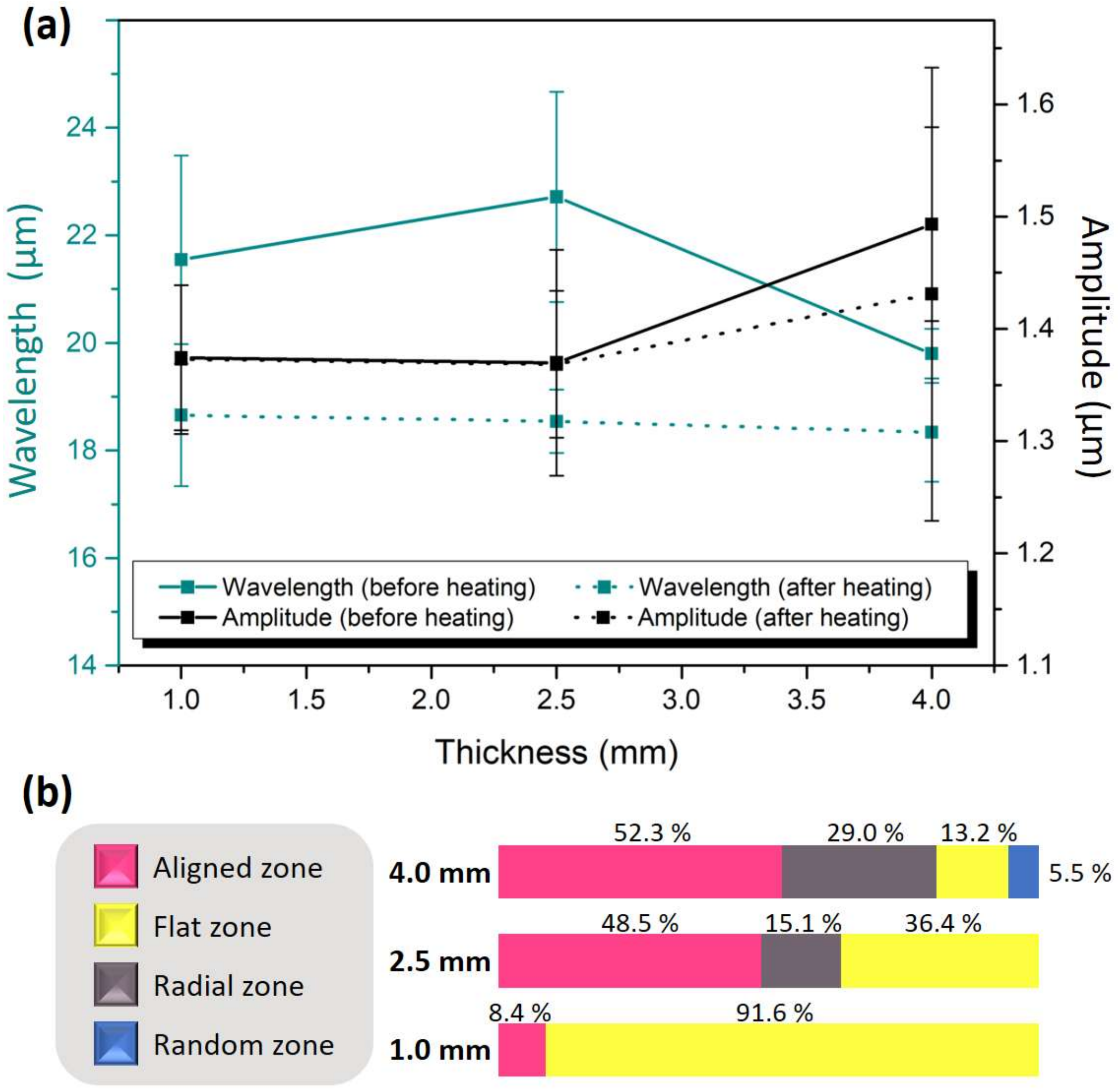

Figure S3. (a) Wavelengths and amplitudes of wrinkles produced on CNTs-PDMS elastomers with various thicknesses. The solid and dashed lines represent the results obtained before and after thermal realignment, respectively. (b) Distribution ratios of the aligned zone, flat zone, radial zone, and random zone in CNTs-PDMS elastomers with various thicknesses.

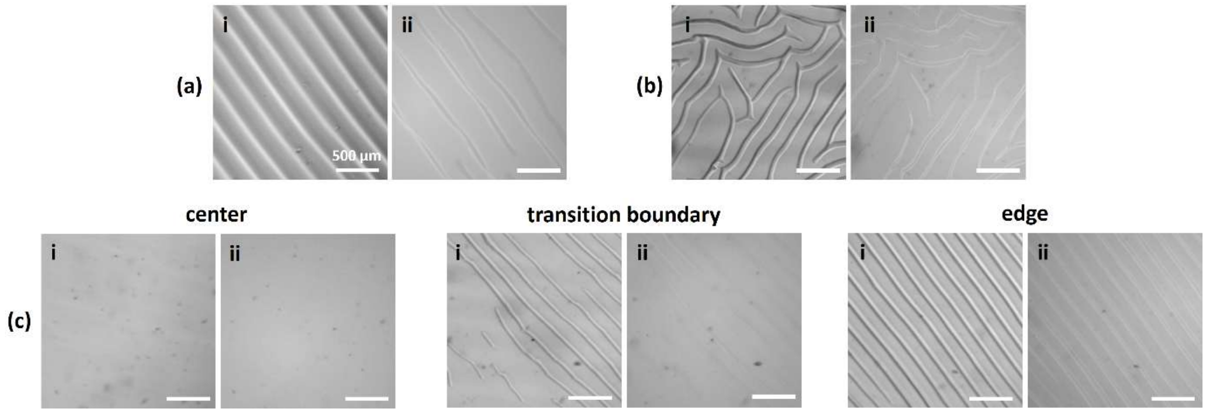

Figure S4. Optical micrographs of the interfacial contact between PS/PDMS wrinkled surfaces and a flat glass slide, showing (i) before adhesion and (ii) under adhesion. Three types of wrinkle morphologies are compared: (a) aligned, (b) random, and (c) realigned wrinkles. For the realigned sample in (c), images are provided at the center, the transition boundary (from the flat area to the aligned area), and the edge. Scale bars represent 500 µm.

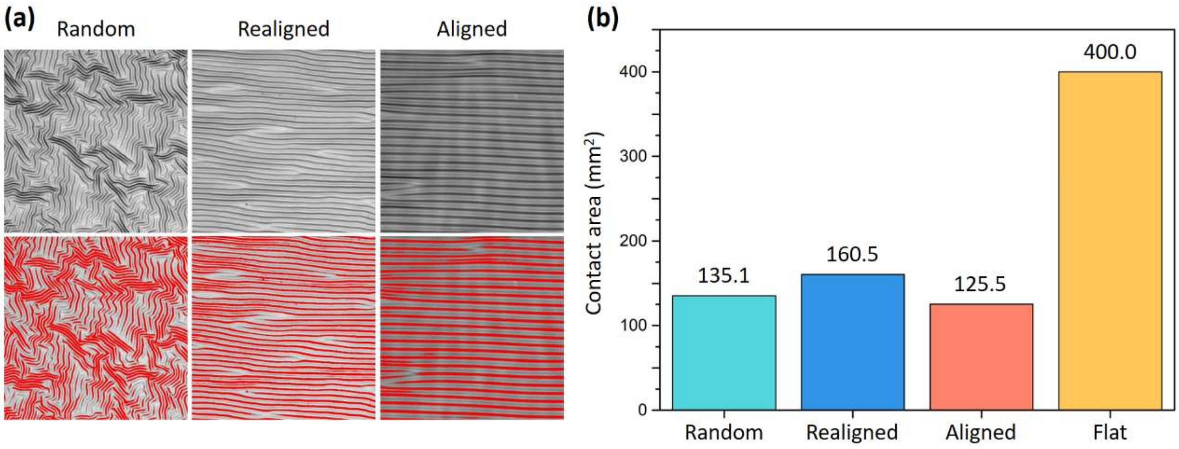

Figure S5. (a) Top: Confocal images of three types of wrinkles. Bottom: Binary images obtained through thresholding, with the red regions indicating the calculated contact areas. (b) Contact areas of surfaces with random wrinkles, realigned wrinkles, aligned wrinkles, and no wrinkles (i.e., a flat surface).

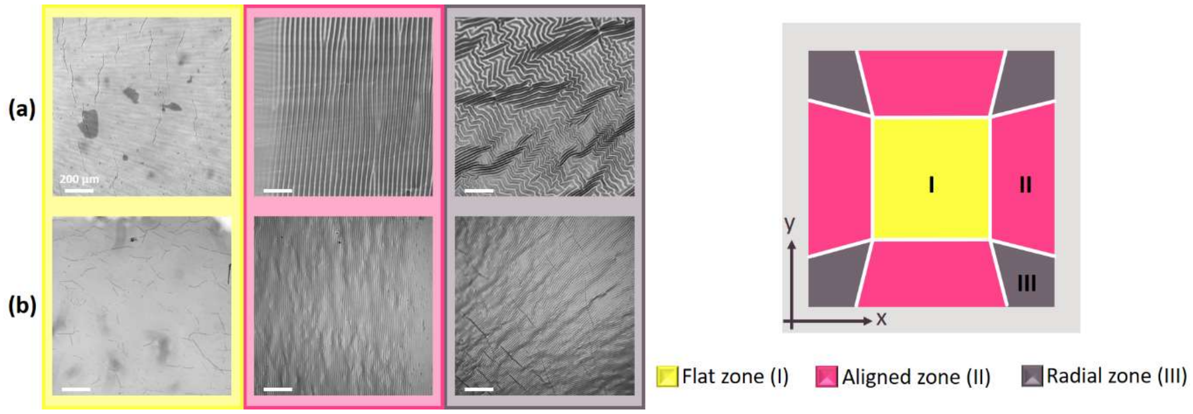

Figure S6. Confocal images of (a) PMMA/PDMS and (b) PS/epoxy wrinkled surface after a heating cycle. Scale bars represent 200 μm.
